# The Gut Microbiota Is Associated with Clearance of Clostridium difficile Infection Independent of Adaptive Immunity

**DOI:** 10.1128/mSphereDirect.00698-18

**Published:** 2019-01-30

**Authors:** Jhansi L. Leslie, Kimberly C. Vendrov, Matthew L. Jenior, Vincent B. Young

**Affiliations:** a Department of Microbiology and Immunology, University of Michigan Medical School, Ann Arbor, Michigan, USA; b Division of Infectious Diseases, Department of Internal Medicine, University of Michigan Medical School, Ann Arbor, Michigan, USA; Carnegie Mellon University; Loyola University Chicago; University of Oregon

**Keywords:** *Clostridium difficile*, adaptive immunity, colonization resistance, intestinal colonization, microbiota

## Abstract

*Clostridium* (*Clostridioides*) *difficile*, a Gram-positive, anaerobic bacterium, is the leading single cause of nosocomial infections in the United States. A major risk factor for Clostridium difficile infection (CDI) is prior exposure to antibiotics, as they increase susceptibility to CDI by altering the membership of the microbial community enabling colonization. The importance of the gut microbiota in providing protection from CDI is underscored by the reported 80 to 90% success rate of fecal microbial transplants in treating recurrent infections. Adaptive immunity, specifically humoral immunity, is also sufficient to protect from both acute and recurrent CDI. However, the role of the adaptive immune system in mediating clearance of C. difficile has yet to be resolved. Using murine models of CDI, we found that adaptive immunity is dispensable for clearance of C. difficile. However, random forest analysis using only two members of the resident bacterial community correctly identified animals that would go on to clear the infection with 66.7% accuracy. These findings indicate that the indigenous gut microbiota independent of adaptive immunity facilitates clearance of C. difficile from the murine gastrointestinal tract.

**IMPORTANCE** Clostridium difficile infection is a major cause of morbidity and mortality in hospitalized patients in the United States. Currently, the role of the adaptive immune response in modulating levels of C. difficile colonization is unresolved. This work suggests that the indigenous gut microbiota is a main factor that promotes clearance of C. difficile from the GI tract. Our results show that clearance of C. difficile can occur without contributions from the adaptive immune response. This study also has implications for the design of preclinical studies testing the efficacy of vaccines on clearance of bacterial pathogens, as inherent differences in the baseline community structure of animals may bias findings.

## Abstract (precis)

Clostridium difficile infection is a major cause of morbidity and mortality in hospitalized patients in the United States. Currently, the role of the adaptive immune response in modulating levels of C. difficile colonization is unresolved. This work suggests that the indigenous gut microbiota is a main factor that promotes clearance of C. difficile from the GI tract. Our results show that clearance of C. difficile can occur without contributions from the adaptive immune response. This study also has implications for the design of preclinical studies testing the efficacy of vaccines on clearance of bacterial pathogens, as inherent differences in the baseline community structure of animals may bias findings.

## INTRODUCTION

Human disease due to anaerobic bacterium *Clostridium* (*Clostridioides*) *difficile* is a significant cause of morbidity and mortality in the United States with an estimated 500,000 cases in the United States yearly (1). A major risk factor for Clostridium difficile infection (CDI) is prior exposure to antibiotics (2). Antibiotics increase susceptibility to CDI by altering the membership of the microbial community and thus the metabolome of the gut, enabling colonization (3). Colonization with C. difficile can manifest in a range of clinical syndromes ranging from asymptomatic colonization to inflammatory colitis characterized by diarrhea with abdominal pain and in severe cases, death. In addition to primary infection, one in five patients treated for CDI experiences recurrent disease (1).

Disease is primarily mediated by the production of two toxins, TcdA and TcdB, which are the major virulence factors for C. difficile (4). TcdA and TcdB are large multidomain proteins, which inactivate cellular rho family GTPases via the addition of a glucose molecule (5). Inactivation of these key regulatory proteins in epithelial cells results in disruption of tight junctions and increased paracellular flow and eventually leads to cell death (6, 7).

The importance of the gut microbiota in providing protection from CDI is underscored by the reported 80 to 90% success rate of fecal microbial transplants in preventing recurrent infection (8–10). Other than microbiome-mediated prevention of colonization, adaptive immunity is also sufficient to provide protection from both acute and recurrent CDI likely via antibody-mediated neutralization of C. difficile toxins TcdA and TcdB (11–14). However, the role of the adaptive immune system in modulating C. difficile colonization has yet to be resolved.

In this study, we sought to determine whether adaptive immunity plays a role in clearance of C. difficile colonization. We found that clearance of C. difficile can occur in the absence of adaptive immunity. Furthermore, the indigenous microbial community membership that exists prior to antibiotic administration and infection was predictive of which animal went on to clear the infection.

## RESULTS

### Clearance of C. difficile can occur in the absence of adaptive immune responses.

**FIG 1:**
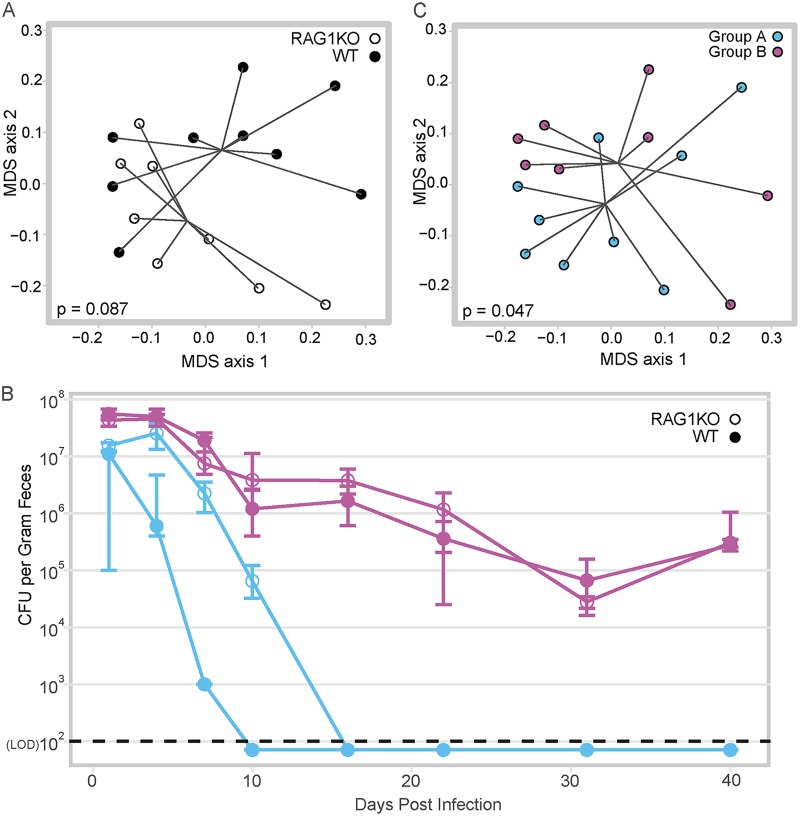
Adaptive immunity is not required for clearance of C. difficile. (A) Multidimensional scaling (MDS) plot of Bray-Curtis dissimilarity between the fecal microbiota of WT mice versus RAG1^−/−^ mice during cohousing, before antibiotic pretreatment. Each circle represents the fecal microbial community from one mouse. Closed circles depict WT mice, while open circles depict RAG1^−/−^ (RAG1 knockout [RAG1KO]) mice; the communities are not significantly different by ANOSIM (*P* = 0.087). (B) Temporal C. difficile colonization by cage. The circles indicate the median level of colonization within a cage, while the bars indicate the interquartile ranges. Groups of mice that were cohoused together are denoted by shared color; closed circles are WT mice, while open circles are RAG1^−/−^ mice. Day 40 colonization was used to determine whether C. difficile CFU/g feces was statically different between the groups (purple WT versus purple RAG1^−/−^ *P* = 0.886, purple WT versus blue RAG1^−/−^ *P* = 0.026, purple RAG1^−/−^ versus blue RAG1^−/−^ *P* = 0.026, purple RAG1^−/−^ versus blue WT *P* = 0.026, purple WT versus blue WT *P* = 0.026). The LOD was 100 CFU/g feces. In cases where no CFU were detected, results are plotted below the LOD for visual clarity, while a value equal to the $\text{LOD}/\sqrt{2}$ was used for statistical tests. Statistical significance was calculated using a Wilcoxon test with Benjamini-Hochberg correction for multiple comparisons. (C) MDS plot of Bray-Curtis dissimilarity of WT versus RAG1^−/−^ mice during cohousing, before antibiotic pretreatment, analyzed by cohousing group rather than genotype. Each circle represents the fecal microbial community from one mouse; the mice that will go on to clear C. difficile are indicated with blue circles versus mice that will remain colonized indicated with purple circles (ANOSIM, *P* = 0.047).

We sought to determine the contribution of adaptive immunity in clearance of C. difficile. To test this, we compared C. difficile infection in wild-type (WT) mice to RAG1^−/−^ mice, which lack both B and T cells. As the two genotypes of mice were derived from separate colonies and others have reported that the microbial community of RAG1^−/−^ mice is distinct from that of WT mice, we cohoused the RAG1^−/−^ mice with WT mice for more than 3 weeks. Cohousing normalized the WT and RAG1^−/−^ mouse fecal communities such that they were not significantly different (ANOSIM *P* = 0.087) (Fig. 1A). Both groups of mice were pretreated with antibiotics, separated into cages based on genotype, and then challenged with C. difficile strain 630. Although all mice were initially colonized, within 3 weeks of challenge, animals in two cages cleared C. difficile, while the remaining animals were persistently colonized (Fig. 1B). Notably, the mice that cleared the infection were WT and RAG1^−/−^. Clearance or persistence colonization with C. difficile did not correspond with the genotype but with the cohousing group. Reanalyzing the preantibiotic microbial communities by cohousing group rather than genotype, we found that the mice that eventually cleared C. difficile had significantly distinct community compared to the mice that remained colonized (ASOSIM *P* = 0.047) (Fig. 1C). These results demonstrate that clearance of C. difficile can occur independently of adaptive immunity.

### Reconstitution of IgG antitoxin antibody is not sufficient to clear C. difficile.

To mitigate any effect of inherent baseline differences in the microbiota of WT and RAG1^−/−^ mice, we tested whether adaptive immunity is sufficient to clear C. difficile by reconstituting RAG1^−/−^ mice with splenocytes from WT mice. Reports of immunization with various C. difficile antigens suggest that antibodies to these antigens may decrease colonization so we additionally tested whether transfer of cells from mice immunized via natural infection with C. difficile might facilitate clearance (15, 16). Splenocytes were collected from WT mice that were either naive or colonized with C. difficile strain 630 for 3 weeks (see Fig. S1A in the supplemental material). Development of humoral immune responses to C. difficile in the donor mice was confirmed by the detection of high titers of anti-TcdA IgG in serum, while uninfected mice had undetectable levels of anti-TcdA serum IgG (*P* < 0.01) (Fig. S1B).

10.1128/mSphereDirect.00698-18.1 FIG S1 Colonization of C. difficile in wild-type donor mice. (A) Temporal colonization of C. difficile by cage. Circles represent the median values (CFU/g feces) for each cage, while the error bars denote upper and lower quartiles. Gray symbols represents the cage of mock-infected mice that were never colonized, while black symbols represent the cage of infected mice. The black dashed line represents the limit of detection, which was 100 CFU/g feces. In cases where no CFU were detected, results are plotted below the LOD line for visual clarity. (B) Anti-TcdA IgG titers in wild-type donor mice (*P* = 0.009). Data from one infected mouse is not shown due to a technical error while measuring the titer of IgG against TcdA. Download FIG S1, TIF file, 0.5 MB. Copyright © 2019 Leslie et al. 2019 Leslie et al. This content is distributed under the terms of the Creative Commons Attribution 4.0 International license.

Recipient RAG1^−/−^ mice were infected with C. difficile strain 630 prior to the adoptive transfer. Donor splenocytes were administered to the recipient RAG1^−/−^ mice 2 days after C. difficile challenge, when C. difficile colonization had already reached high levels. Recipient mice were randomly assigned to one of three groups and either received splenocytes from naive WT donors, splenocytes from infected WT donors, or vehicle.

**FIG 2:**
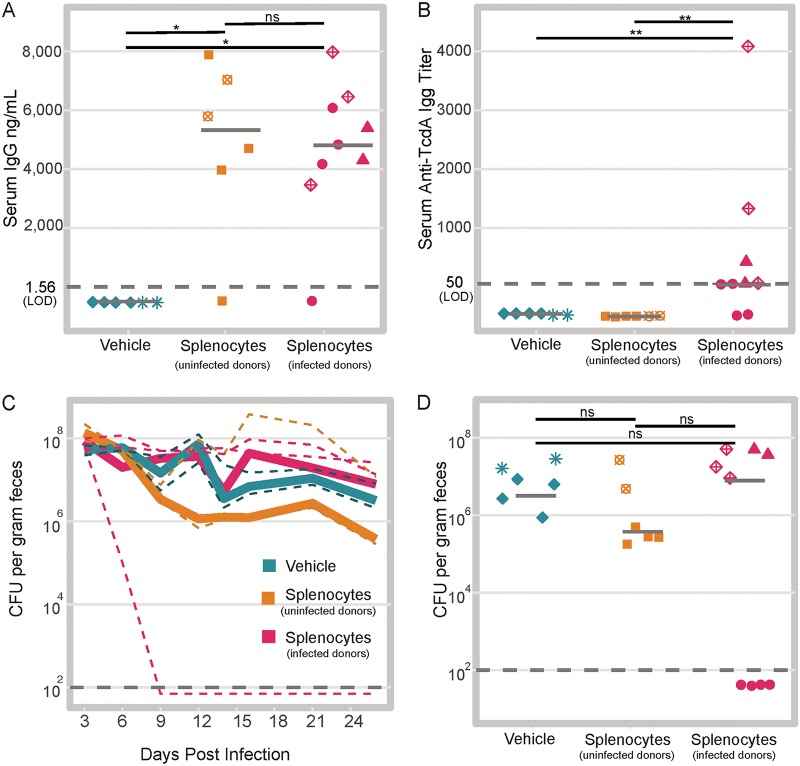
Adoptive transfer of WT splenocytes into RAG1^−/−^ mice is not sufficient to promote clearance. (A) Total serum IgG in the recipient RAG1^−/−^ mice 24 days after injection of splenocytes (mice that received vehicle versus uninfected donor splenocytes *P* = 0.014; mice that received vehicle versus infected donor splenocytes *P* = 0.011; mice that received uninfected donor splenocytes versus infected donor splenocytes *P* = 0.814). Shapes represent mice in the same cage. Note two mice that were given splenocytes did not develop detectable serum IgG. Each symbol represents the value for an individual mouse. Solid gray lines represent the median values for groups. ns, not significant. (B) Anti-TcdA IgG titers in recipient RAG1^−/−^ mice 24 days after transfer of splenocytes (mice that received vehicle versus infected donor splenocytes *P* = 0.008; mice that received vehicle versus uninfected donor splenocytes *P* = not significant; mice that received uninfected donor splenocytes versus infected donor splenocytes *P* = 0.008). Solid gray lines represent the medians. (C) Time course of intestinal colonization levels with C. difficile colored by treatment group. Solid lines represent the median values (CFU of C. difficile per gram of feces) for treatment groups. Dashed lines represent median colonization values within cages. (D) Colonization on day 26 postinfection (day 24 after adoptive transfer) colored by treatment group (mice that received vehicle versus uninfected donor splenocytes *P* = 0.689; mice that received vehicle versus infected donor splenocytes *P* = 1; mice that received uninfected donor splenocytes versus infected donor splenocytes *P* = 1). Solid gray lines represent the median values. The dark gray dashed line in each panel represents the limit of detection (LOD). In cases where no CFU were detected, results are plotted below the LOD for visual clarity, while a value equal to the $\text{LOD}/\sqrt{2}$ was used for statistical tests. Statistical significance was calculated using a Wilcoxon test with Benjamini-Hochberg correction for multiple comparisons.

To confirm engraftment of the WT cells, we measured total serum IgG in the recipient mice 3 weeks after transfer. The mice that received splenocytes had significantly higher levels of total serum IgG posttransfer compared to the mice that received vehicle (*P* < 0.05) (Fig. 2A). Of the mice that received splenocytes, two did not develop any detectable serum IgG. There was no difference in the levels of total serum IgG between the mice that received splenocytes from infected donors versus uninfected donors (*P* > 0.05). Furthermore, we determined that we successfully transferred anti-C. difficile immunity, as we detected anti-TcdA IgG only in the sera from mice that received splenocytes from the infected donors (*P* < 0.01) (Fig. 2B).

After adoptive transfer, the levels of C. difficile in the feces were monitored for 3 weeks. We observed clearance of C. difficile from one cage of mice in the group that received splenocytes from infected donors. However, clearance of C. difficile did not occur in any of the other animals within that treatment group (Fig. 2C). Three weeks after transfer, there was no significant difference in the levels of colonization in any of the treatment groups (Fig. 2D). Notably, in the cage with mice that cleared C. difficile, one mouse had undetectable levels of serum IgG, while the other three mice in the cage had detectable levels (Fig. 2A, filled pink circles). Together these results suggest that reconstitution of adaptive immunity is not sufficient for clearance of C. difficile.

**FIG 3:**
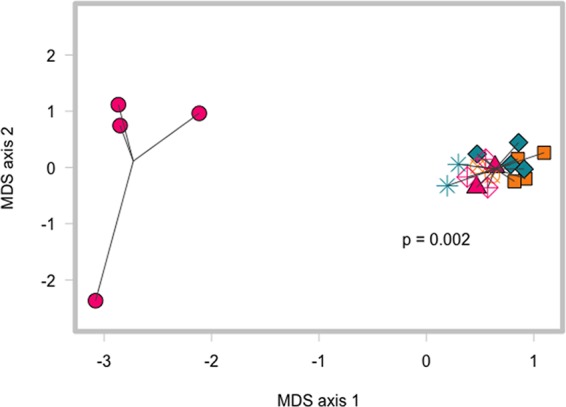
Clearance of C. difficile colonization is associated with significantly different pretransfer gut microbiota, not treatment groups. Multidimensional scaling (MDS) plot of Bray-Curtis dissimilarity index comparing communities in mice at day 1 postinfection (before adoptive transfer). Cages that cleared infection are shown as pink circles, while the other cages are represented by different shapes (mice that went on to clear C. difficile versus all other mice, ANOSIM *P* = 0.002).

The range in the levels of colonization we observed within each treatment group suggested that adaptive immunity is not sufficient to explain the differences in clearance of C. difficile. Visualization of the Bray-Curtis dissimilarity between the day 1 postinfection communities (before the adoptive transfer) using multidimensional scaling revealed that the mice that went on to clear C. difficile had a distinct community compared to the mice that would remain colonized by C. difficile (ANOSIM *P* = 0.002) (Fig. 3). This result suggests the structure of gut microbiota, rather than adoptive transfer of splenocytes, is associated with clearance of C. difficile.

### Specific members of the microbiota are altered in mice with reconstituted adaptive immunity.

**FIG 4:**
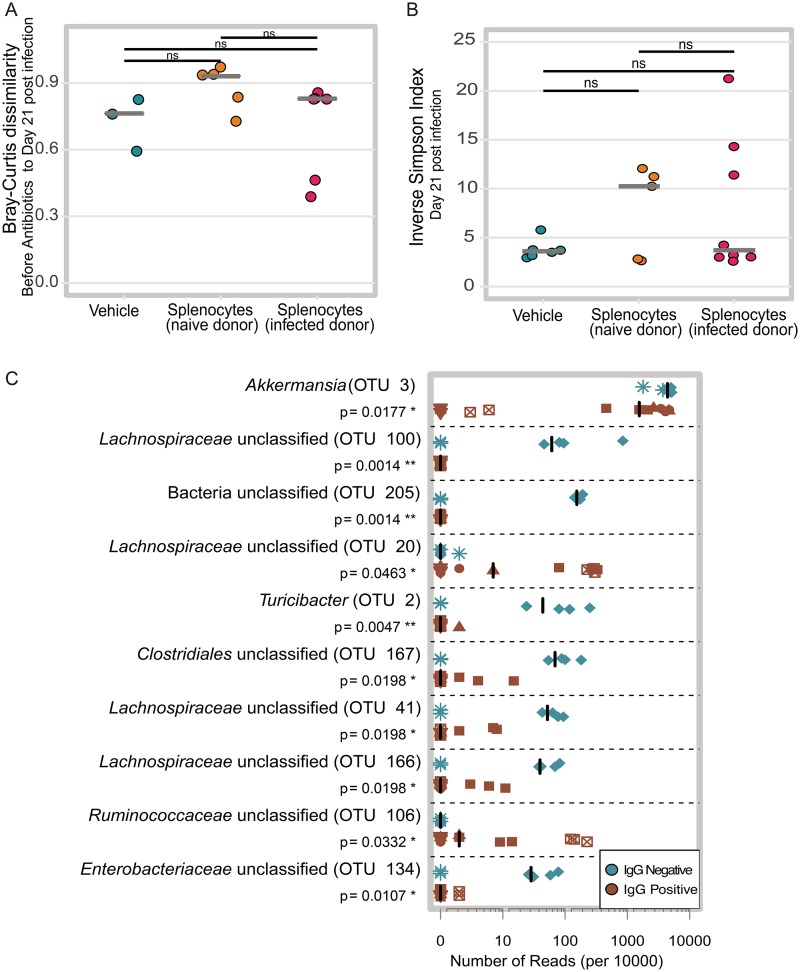
Effect of reconstitution of adaptive immunity on the microbiota. (A) The Bray-Curtis dissimilarity between each mouse’s preantibiotic and day 21 postinfection communities (mice that received uninfected donor splenocytes versus infected donor splenocytes *P* = 0.214, mice that received uninfected donor splenocytes versus vehicle *P* = 0.214, mice that received infected donor splenocytes versus vehicle *P* = 0.714). Solid gray lines represent the median values for groups. (B) Inverse Simpson diversity of communities day 21 postinfection communities (mice that received uninfected donor splenocytes versus infected donor splenocytes *P* = 0.9433, mice that received uninfected donor splenocytes versus vehicle *P* = 0.943, mice that received infected donor splenocytes versus vehicle *P* = 0.943). Solid gray lines represent the medians. (C) Relative abundance of the top 10 OTUs with the highest LDA distinguishing between vehicle-treated RAG1^−/−^ mice and IgG-positive mice. Each symbol represents the value for a single mouse. The different shapes represent different cages. Solid black lines represent the medians. Statistical significance was calculated using a Wilcoxon test with Benjamini-Hochberg correction for multiple comparisons.

The microbiota and the immune system have been previously shown to modulate one another through numerous complex interactions (17, 18). In the cefoperazone mouse model of infection, the diversity of microbiota begins to increase by 2 weeks following cessation of the antibiotic (19). Therefore, we asked whether reconstitution of adaptive immunity altered the recovery of the community following antibiotics and infection with C. difficile. We examined the gut microbial community structure of the mice over the course of the experiment using 16S rRNA gene amplicon sequencing. Our first approach sought to determine whether we could detect changes in the overall microbial community composition of the mice. We calculated the Bray-Curtis dissimilarity between each mouse’s day 21 sample (19 days after the adoptive transfer) and their preantibiotic sample. We hypothesized that reconstitution of adaptive immunity might prevent the microbiota from returning to the same structure as was observed before adoptive transfer. Thus, we thought that perhaps the mice that received splenocytes might have higher Bray-Curtis dissimilarity values compared to the mice that received only vehicle. Since we were unable to confirm that we successfully restored adaptive immune function in two mice that received splenocytes (Fig. 2A), we excluded them from the rest of analysis as our questions hinged on immune status-gut microbiota interactions. Additionally, we lost the ability to calculate this metric for a couple of mice due to the lack of preantibiotic samples. Comparing the Bray-Curtis dissimilarity results between the three treatment groups revealed no significant differences between any of the groups (Fig. 4A). We also wondered whether the addition of adaptive immunity might alter alpha diversity, so we calculated the inverse Simpson index for each fecal community at day 19 posttransfer (day 21 postinfection). We did not observe any significant differences between the treatment groups by this metric (Fig. 4B). This suggested that by broad evaluations of community structure, the perturbation of antibiotics and infection with C. difficile potentially has a much greater effect on the microbial community than any effects due to immune reconstitution.

While we saw no significant differences in the recovery of the community structure or alpha diversity at day 21 postinfection, we wondered whether perhaps the levels of only a few operational taxonomic units (OTUs) were altered by reconstitution of the adaptive immune system. For this analysis, we grouped all of the mice that received splenocytes and developed detectable levels of serum IgG at day 26 postinfection together and called them the IgG-positive mice. The mice that received only vehicle and thus had undetectable levels of serum IgG were designated the IgG-negative mice. Using OTU abundance from day 21 postinfection samples, linear discriminant analysis effect size (LefSe) identified 27 OTUs with linear discriminant analysis (LDA) values greater than two. The 10 OTUs with the highest LDA values were primarily enriched in the IgG-negative mice (Fig. 4C). OTU 3, which is classified as *Akkermansia,* had the highest LDA value. This OTU was found at a significantly lower abundance in the IgG-positive mice than in the IgG-negative mice. A decrease in *Akkermansia* after reconstitution of adaptive immunity via transfer of bone marrow from wild-type mice into RAG1^−/−^ mice has been reported by another group (20). While the decrease in OTU 3 in the IgG-positive mice was observed across cages, many of the other OTUs that discriminated between the IgG-positive and -negative mice were detected in only one of the cages with IgG-negative mice.

### Random forest feature selection identifies OTUs in the preantibiotic community that differentiates mice that will remain persistently colonized versus clear.

**FIG 5:**
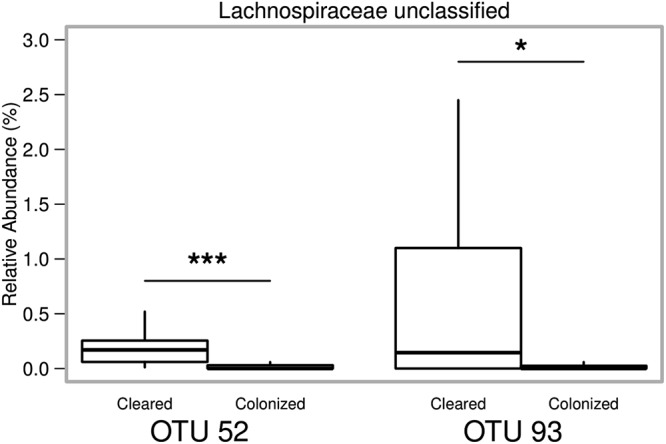
Relative abundance of OTU pretreatment that correctly classify mice that will go on to clear C. difficile infection. Box and whisker plots showing the relative abundance of the two OTUs from the pretreatment fecal microbiota that differentiate animals that will go on to clear C. difficile infection with 66.6% accuracy. Statistical significance was calculated using a Wilcoxon test with Benjamini-Hochberg correction.

After our previous analyses, we made the consistent observation that structure of the gut microbiome was associated with clearance of C. difficile, even prior to antibiotic treatment (Fig. 1C). We questioned whether specific OTUs present in the mice before any intervention may have differentiated mice that would go on to clear the infection. For this analysis, we pooled data from three independent experiments (the two described earlier and a third experiment including only WT mice) where cages of mice had spontaneously cleared C. difficile (Fig. S2). We utilized random forest for feature selection to identify OTUs that could classify mice as “cleared” or “colonized” based on their preintervention microbiota. Using the entire pretreatment community, we could classify the mice as “cleared” or “colonized” with 76.9% accuracy. However, this model was better at classifying mice that would remain colonized and was poor at classifying mice that would go onto clear C. difficile, with an accuracy of only 25%. Nine out of the top 10 OTUs that most contributed to classification were from the *Firmicutes* phylum (Fig. S3). Two OTUs in particular (OTUs 52 and 93) ranked highest in their ability to discriminate between the groups and were significantly increased in abundance in mice that would go on to clear *C*. *difficile*. Therefore, we tested whether those two OTUs alone were sufficient to classify the mice. Generating a new random forest model using only those two OTUs, we found that the overall model improved to 82.9% accuracy in classification. Furthermore, these two OTUs could correctly classify mice that would go on to clear C. difficile with 66.6% accuracy (Fig. 5).

10.1128/mSphereDirect.00698-18.2 FIG S2 Colonization of C. difficile in wild-type mice included in random forest analysis. Temporal colonization of C. difficile by cage. Circles represents the median values (CFU/g feces) for each cage, while the error bars denote upper and lower quartiles. Colors represent the different cages of mice. The black dashed line represents the limit of detection, which was 100 CFU/g feces. In cases where no CFU were detected, results are plotted below the LOD line for visual clarity. Download FIG S2, TIF file, 0.3 MB. Copyright © 2019 Leslie et al. 2019 Leslie et al. This content is distributed under the terms of the Creative Commons Attribution 4.0 International license.

10.1128/mSphereDirect.00698-18.3 FIG S3 Intact community predicts outcome of *C*. *difficile* infection. (Left) Top 10 OTUs with highest mean decrease in accuracy from the random forest classifier using data from the whole community. (Right) Relative abundance of OTUs from the left panel. Black bars represent the median values. Statistical significance was calculated using a Wilcoxon test with Benjamini-Hochberg correction. Download FIG S3, TIF file, 1.8 MB. Copyright © 2019 Leslie et al. 2019 Leslie et al. This content is distributed under the terms of the Creative Commons Attribution 4.0 International license.

## DISCUSSION

In this study, we asked whether adaptive immunity was required for clearance of the gastrointestinal pathogen C. difficile. Results from multiple experimental models lead us to conclude that clearance of C. difficile in mice can occur without contributions from adaptive immune responses. This finding is in contrast to the paradigm observed in other gastrointestinal infections. For example, infection with the attaching-effacing pathogen Citrobacter rodentium provides a framework by which the adaptive immunity facilitates clearance (21, 22). In addition to the potential direct effects, adaptive immunity may have on the bacterium itself, it is known that there is a complex interaction loop between the microbiota and host immune response. Both the innate and adaptive arms of the immune system regulate membership of the gut microbial community, while the gut microbiota in turn modulates the immune system via the production of metabolites and/or MAMPs (23).

Our results show that reconstitution of adaptive immunity is associated with altered abundance of some bacteria in the gut; however, it does not impact levels of C. difficile colonization. We found that in the reconstituted RAG1^−/−^ mice that developed serum IgG, there was a decreased abundance of *Akkermansia* (OTU 3). Another group has previously observed this result; however, we were surprised to see the same trend in our model, as our mice were also subjected to antibiotic therapy and infection with C. difficile (20). In the two mice that received splenocytes but did not have detectable serum IgG, the abundance of the *Akkermansia* OTU was very low (see Fig. S4A in the supplemental material). There are numerous reasons why this could be the case, the first being that a lack of serum IgG does not preclude successful transfer of T cells which may be responsible for modulating levels of *Akkermansia* in wild-type mice. Additionally, fecal IgG or IgA from the mice that had successful transfers may have been transmitted via coprophagy in sufficient quantities to modulate the levels of *Akkermansia* in the IgG-negative mice that were sharing their cage. Since the relative abundance of OTU 3 was not significantly different between the groups in the pretreatment samples, we can conclude that the differences we observed were a result of the experimental conditions and not merely baseline differences in their microbiota (Fig. S4B). *Akkermansia* has been implicated in the modulation of health processes such as regulation of host metabolism, so further studies are necessary to fully elucidate the factors that regulate its abundance in the gut (24, 25).

10.1128/mSphereDirect.00698-18.4 FIG S4 *Akkermansia* (OTU 3) relative abundance. (A) Relative abundance of OTU 3 in all the mice on day 21 postinfection in the adoptive transfer experiment. Mice that received splenocytes but did not develop detectable total serum IgG have low levels of this OTU, unlike the mice that received vehicle. There is a significant difference in the abundance of OTU 3 in the mice that received vehicle compared to the mice that had successful transfers of WT splenocytes (*P* = 0.0199) by Wilcoxon test. (B) Relative abundance of OTU 3 before any treatment. Download FIG S4, TIF file, 0.5 MB. Copyright © 2019 Leslie et al. 2019 Leslie et al. This content is distributed under the terms of the Creative Commons Attribution 4.0 International license.

On the basis of our repeated observations that altered communities early in the experimental timeline were associated with clearance of C. difficile, we used random forest to eventually identify just two OTUs that could classify mice that would go on to clear C. difficile with 66.6% accuracy. Previous work using a similar approach identified OTUs present on the day of challenge that were predictive of levels of colonization on day 1 postinfection; however, we are the first group to assess whether the composition of the murine gut microbiota before any treatment might affect the outcome of C. difficile infection (26). Both of the OTUs we identified belong to the family *Lachnospiraceae* and were enriched in mice that would go on to clear C. difficile infection. Our group has previously observed that high levels of *Lachnospiraceae* are associated with protection from severe disease in a murine model of CDI (27). One possibility is that these bacteria are just inherently resistant to cefoperazone; however, *in vitro* antibiotic susceptibility testing of *Lachnospiraceae* isolates from our mouse colony suggest that this is not the case (data not shown). Furthermore, we have also reported that monoassociation of germfree mice with a single *Lachnospiraceae* isolate partially restored colonization resistance (28). It is tempting to speculate multiple *Lachnospiraceae* isolates might be able to fully restore colonization resistance. However, it remains to be seen whether the same mechanisms, which prevent initial colonization of C. difficile, play a role in clearance of C. difficile.

Our results suggest that community resilience is intrinsic to the community membership at baseline, prior to any antibiotic treatment. Additionally, these data suggest the possibility of predicting individuals that will be at risk for persistent colonization before antibiotic therapy. However, a crucial first step is to determine whether predictive OTUs are different across perturbations such as various classes of antibiotic therapy. Finally, our findings have implications for the design of future preclinical studies testing the efficacy of vaccines or other manipulations of adaptive immunity on the level of colonization as “cage effects,” or inherent differences in the baseline community structure of animals within cages may bias findings. Experimental approaches that can be implemented to account for the role of the microbiota include cohousing, using multiple cages for each experimental condition, and the use of littermate controls (29).

## MATERIALS AND METHODS

### Animal husbandry.

Both male and female specific-pathogen-free (SPF) C57BL/6 mice aged 5 to 12 weeks were used in these studies. The wild-type (WT) mice were from a breeding colony at the University of Michigan, originally derived from Jackson Laboratories over a decade ago. The RAG1^−/−^ (B6.129S7-*Rag1^tm1Mom^*/J) mice were from a breeding colony started with mice from Jackson Laboratories in 2013. Animals were housed in filter top cages with corn cob bedding and nestlet enrichment. Water bottles were autoclaved empty and filled in a biological safety cabinet with either sterile water or antibiotic dissolved in sterile water. Mice were fed a standard irradiated chow (LabDiet 5LOD) and had access to food and water *ad libitum*. Cage changes were carried out in a biological safety cabinet. The frequency of cage changes varied depending on the experiment. To prevent cross-contamination between cages, hydrogen peroxide-based disinfectants in addition to frequent glove changes were utilized during all manipulation of the animals. The mice were maintained under 12 h of light/dark cycles in facilities maintained at a temperature of 72°C ± 4°C. Animal sample size was not determined by a statistical method. Multiple cages of animals for each treatment were used to control for possible differences in the microbiota between cages. Mice were evaluated daily for signs of disease. Euthanasia was carried out via CO_2_ asphyxiation when mice were determined to be moribund or at the conclusion of the experiment. Animal studies were conducted under the approval of The University of Michigan Committee on the Care and Use of Animals; husbandry was performed in an AAALAC-accredited facility.

### Spore preparation.

Spore stocks of C. difficile strain 630 (ATCC BAA-1382) were prepared as previously described with the following modifications; strains were grown overnight in 5 ml of Difco Columbia broth (BD Biosciences catalog no. 294420), which was added to 40 ml of Clospore medium (3, 30).

### Infections.

In experiments comparing colonization in WT and RAG1^−/−^ mice, age- and sex-matched mice were cohoused for 33 days starting at 3 weeks of age and continuing through cefoperazone administration. Upon infection, animals were separated into single genotype housing. Mice were made susceptible to infection by providing drinking water with the addition of 0.5 mg/ml cefoperazone (MP Pharmaceuticals catalog no. 0219969501) in Gibco distilled water (catalog no. 15230147) for the mice to drink *ad libitum*. The antibiotic water was changed every 2 days and was provided for 10 days. After 2 days of supplying drinking water without antibiotic, the mice were challenged with either spores or water (mock). C. difficile spores suspended in 50 μl of Gibco distilled water were administered via oral gavage. The number of viable spores in each inoculum was enumerated by plating for CFU per milliliter on prereduced taurocholate cycloserine cefoxitin fructose agar (TCCFA). TCCFA was made as originally described (31) with the following modifications. The agar base consisted of 40 g of Proteose Peptone No. 3 (BD Biosciences catalog no. 211693), 5 g of Na_2_HPO_4_ (Sigma-Aldrich catalog no. S5136), 1 g of KH_2_PO_4_ (Fisher catalog no. P285500), 2 g NaCl (J.T. Baker catalog no. 3624-05), 0.1 g MgSO_4_ (Sigma catalog no. M7506), 6 g fructose (Fisher catalog no. L95500), and 20 g of agar (Life Technologies catalog no. 30391-023) dissolved in 800 ml of Milli-Q water. After adjusting the volume to 1 liter, the medium was autoclaved and supplemented with D-cycloserine (Sigma-Aldrich catalog no. C6880) to a final concentration of 250 μg/ml, cefoxitin to a final concentration of 16 μg/ml (Sigma-Aldrich catalog no. C4786), and taurocholate to a final concentration of 0.1% (Sigma catalog no. T4009). Over the course of the infection, mice were routinely weighed, and stool was collected for quantitative culture. Mice were challenged with between 10^2^ and 10^4^ CFU.

### Quantitative culture.

Fresh voided fecal pellets were collected from each mouse into a preweighed sterile tube. Following collection, the tubes were reweighed and passed into an anaerobic chamber (Coy Laboratories). In the chamber, each sample was diluted 1 to 10 (wt/vol) using prereduced sterile PBS and serially diluted. One hundred microliters of a given dilution was spread onto prereduced TCCFA or when appropriate TCCFA supplemented with a final concentration of either 2 or 6 μg/ml of erythromycin (Sigma catalog no. E0774). Strain 630 is erythromycin resistant; use of erythromycin in TCCFA plates reduced background growth from other bacteria in the sample. The plates were incubated anaerobically at 37°C, and colonies were enumerated at 18 to 24 h. The plates that were used to determine whether mice were negative for C. difficile were kept and rechecked at 48 h.

### Splenocyte recovery and transfer.

Spleens from individual animals were aseptically harvested from donor mice. Following harvest, the spleen was gently homogenized using sterile glass slides to remove the cells from the capsule. Cells were suspended in filter-sterilized RPMI complete medium consisting of RPMI plus l-glutamine (Gibco catalog no. 11875-093) supplemented with 10% FBS (Gibco catalog no. 16140-071), 1% 100× penicillin-streptomycin (Gibco catalog no. 15070-063), 1% 1 M HEPES (Gibco catalog no. 15630-080), 1% 100× nonessential amino acids (Gibco catalog no. 11140-050), 1% 100 mM sodium pyruvate (Gibco catalog no. 11360-070), and 0.05 ml of 1 M 2-mercaptoethanol (Sigma catalog no.M3148). To remove large debris, the cell suspension was filtered through a 40-μm cell strainer. Cells were pelleted by centrifugation at 1,500 rpm for 5 min at 4°C. Following the spin, the pellet was suspended in red blood cell lysing buffer (Sigma catalog no. R7757) and incubated with the solution for no more than 5 min. Lysis was stopped with the addition of RMPI complete medium, and cells were enumerated manually using a hemocytometer. Following enumeration, the cells were pelleted again by centrifugation at 1,500 rpm for 5 min at 4°C and resuspended in Leibovitz’s L-15 (Corning catalog no. 10-045-CV) medium. Recipient mice were injected into the peritoneal cavity with 2 × 10^7^ cells in 0.25 ml of L-15 medium. Mice that received vehicle were injected with 0.25 ml of L-15 medium only.

### Blood collection.

Blood was collected from either the saphenous vein for pretreatment time points or via heart puncture at the experimental endpoint. Collections from the saphenous vein utilized capillary tubes (Sarstedt microvette catalog no. CB300 Z), while blood collected via heart puncture utilized a polymer gel-based separator tube (BD Microtainer SST). Following collection, tubes were spun according to the manufacturer’s instructions, and serum was aliquoted and stored at −80°C until use.

### Total IgG ELISA.

Total serum IgG levels were measured using the IgG (Total) Mouse Uncoated ELISA kit (ThermoFisher Scientific catalog no. 88-50400). Each sample was diluted 500-fold in assay buffer and run in duplicate with Southern Biotech TMB Stop Solution (catalog no. 0412-01) used as the stop solution. Optical density values were measured at 450 nm and 570 nm on a VersaMax plate reader (Molecular Devices, Sunnyvale, CA) and corrected by subtracting the measurement at 570 nm from the measurement at 450 nm. A four-parameter standard curve was used to calculate sample concentration values.

### Anti-C. difficile TcdA IgG ELISA.

Titers of serum IgG specific to C. difficile TcdA (toxin A) was measured by ELISA as previously described (32) with the following modifications. Serum from RAG1^−/−^ mice that received an adoptive transfer was diluted 1:50 in blocking buffer with subsequent serial dilutions to a final dilution of 1:12,150. Serum from the wild-type mice was diluted 1:1,200 in blocking buffer with subsequent serial dilutions to a final dilution of 1:874,800. Each sample was run in duplicate. Each plate had the following negative controls: all reagents except serum and all reagents except toxin and preimmune serum if applicable. Additionally, each plate had a positive control consisting of toxin-coated wells reacted with mouse TcdA monoclonal antibody TGC2 diluted 1:5,000 in blocking buffer (Antibodies Online catalog no. ABIN335169). The optical density at 410 nm and 650 nm was recorded on a VersaMax plate reader (Molecular Devices, Sunnyvale, CA). The absorbance for each sample was corrected by subtracting the OD_650_ reading from the OD_410_ reading. The anti-TcdA IgG titer for each sample was defined as the last dilution with a corrected OD_410_ greater than the average corrected OD_410_ of the negative-control wells plus three times the standard deviation of those wells.

### DNA extraction.

Genomic DNA was extracted from approximately 200 to 300 μl of fecal sample using the MoBio PowerSoil HTP 96 DNA isolation kit (formerly MoBio, now Qiagen) on the Eppendorf EpMotion 5075 automated pipetting system according to the manufacturer’s instructions.

### Sequencing.

The University of Michigan Microbial Systems Laboratory constructed amplicon libraries from extracted DNA as described previously (33). Briefly, the V4 region of the 16S rRNA gene was amplified using barcoded dual index primers as described by Kozich et al. (34). The PCR mixture included the following: 5 μl of 4 μM stock combined primer set, 0.15 μl of Accuprime high-fidelity *Taq* with 2 μl of 10× Accuprime PCR II buffer (Life Technologies catalog no. 12346094), 11.85 μl of PCR-grade water, and 1 μl of template. The PCR cycling conditions were as follows: (i) 95°C for 2 min; (ii) 30 cycles with 1 cycle consisting of 95°C for 20 s, 55°C for 15 s, and 72°C for 5 min; and (iii) 10 min at 72°C. Following construction, libraries were normalized and pooled using the SequelPrep normalization kit (Life Technologies catalog no. A1051001). The concentration of the pooled libraries was determined using the Kapa Biosystems library quantification kit (Kapa Biosystems catalog no. KK4854), while amplicon size was determined using the Agilent Bioanalyzer high-sensitivity DNA analysis kit (catalog no. 5067-4626). Amplicon libraries were sequenced on the Illumina MiSeq platform using the MiSeq reagent 222 kit V2 (catalog no. MS-102-2003) (500 total cycles) with modifications for the primer set. Illumina’s protocol for library preparation was used for 2 nM libraries, with a final loading concentration of 4 pM spiked with 10% genomic PhiX DNA for diversity.

### Sequence curation and analysis.

Raw sequences were curated using the mothur v.1.39.0 software package (35) following the Illumina MiSeq standard operating procedure. Briefly, paired-end reads were assembled into contigs and aligned to the V4 region using the SILVA 16S rRNA sequence database (release v128) (36). Any sequences that failed to align were removed. Sequences that were flagged as possible chimeras by UCHIME were also removed (37). Sequences were classified with a naive Bayesian classifier (38) using the Ribosomal Database Project (RDP) and clustered into operational taxonomic units (OTUs) using a 97% similarity cutoff with the Opticlust clustering algorithm (39).

The number of sequences in each sample was then rarefied to 10,000 sequences to minimize bias due to uneven sampling. For feature selection, the shared file was filtered to remove any OTU that was in less than six samples across the entire data set. The mothur implementation of LefSe (linear discriminant analysis effect size) was used to determine OTUs that differentiated IgG-positive mice versus RAG1^−/−^ mice given vehicle 19 days after adoptive transfer (40). Following curation in mothur, further data analysis and figure generation were carried out in R (v 3.3.3) using standard and loadable packages (41).

Most of the analysis relied on the R package vegan (42). This includes determining the axes for the multidimensional scaling (MDS) plots using Bray-Curtis dissimilarity calculated from sequence abundance. Additionally, vegan was used to determine significance between groups using ANOSIM, calculation of inverse Simpson index, and Bray-Curtis dissimilarity between samples. Final figures were modified and arranged in Adobe Illustrator CC. For the purpose of distinguishing between values that were detected at the limit of detection (LOD) versus those that were undetected, all results that were not detected by a given assay were plotted at an arbitrary point below the LOD. However, for statistical analysis, the value of $\text{LOD/}\sqrt{2}$ was substituted for undetected values. The Wilcoxon rank sum test was used to determine significant differences, and when appropriate, reported *P* values were corrected for multiple comparisons using the Benjamini-Hochberg correction.

### Random forest analysis.

Random forest analysis was performed using R (v.3.2.3) using the randomForest package (43, 44). Model parameters ntree and mtry were tuned based on the input data sets in order to achieve optimal classification without overfitting (45). Briefly, ntree was calculated by multiplying the total number of OTUs included in the analysis by a ratio of the quantity of samples in each classification category. Additionally, mtry was defined as the square root of the number of OTUs. The informative cutoff for mean decease accuracy (MDA) values was determined by the absolute value of the lowest MDA measured (46). Testing for significant differences in OTU relative abundances following feature selection was performed by using the Wilcoxon signed rank test with Benjamini-Hochberg correction.

### Data availability.

The raw paired-end reads of the sequences for all samples used in this study can be accessed in the Sequence Read Archive under accession no. PRJNA388335. The data and code for all analysis associated with this study are available at https://github.com/jlleslie/AdaptiveImmunity_and_Clearance.
