# Supplementary figures and images for: The Gut Microbiota Is Associated with Clearance of Clostridium difficile Infection Independent of Adaptive Immunity
Source: mSphere. 2019 Jan 30;4(1):e00698-18. doi: 10.1128/mSphereDirect.00698-18 (PMC6354811; doi:10.1128/mSphereDirect.00698-18)

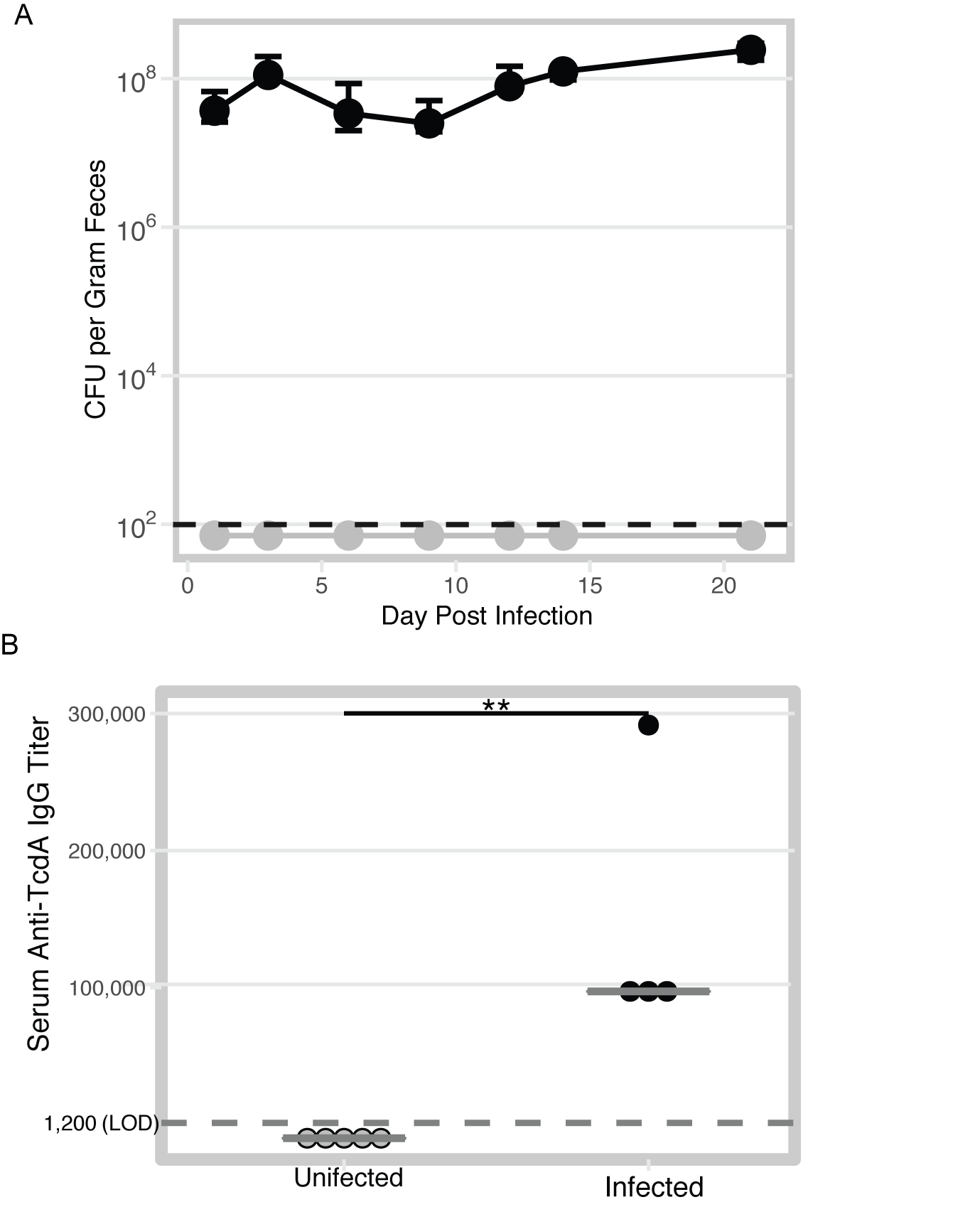

Supplement: FIG S1 [file mSphereDirect.00698-18-sf001.tif]

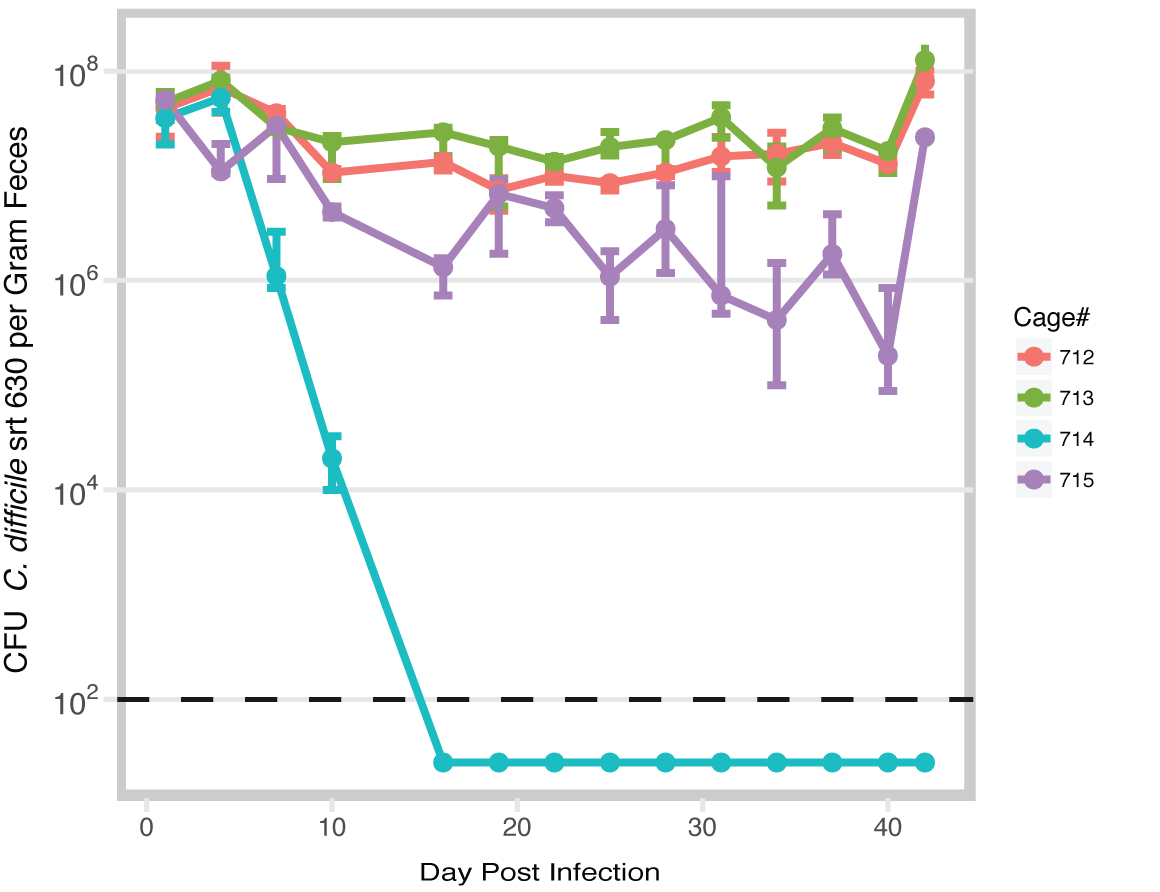

Supplement: FIG S2 [file mSphereDirect.00698-18-sf002.tif]

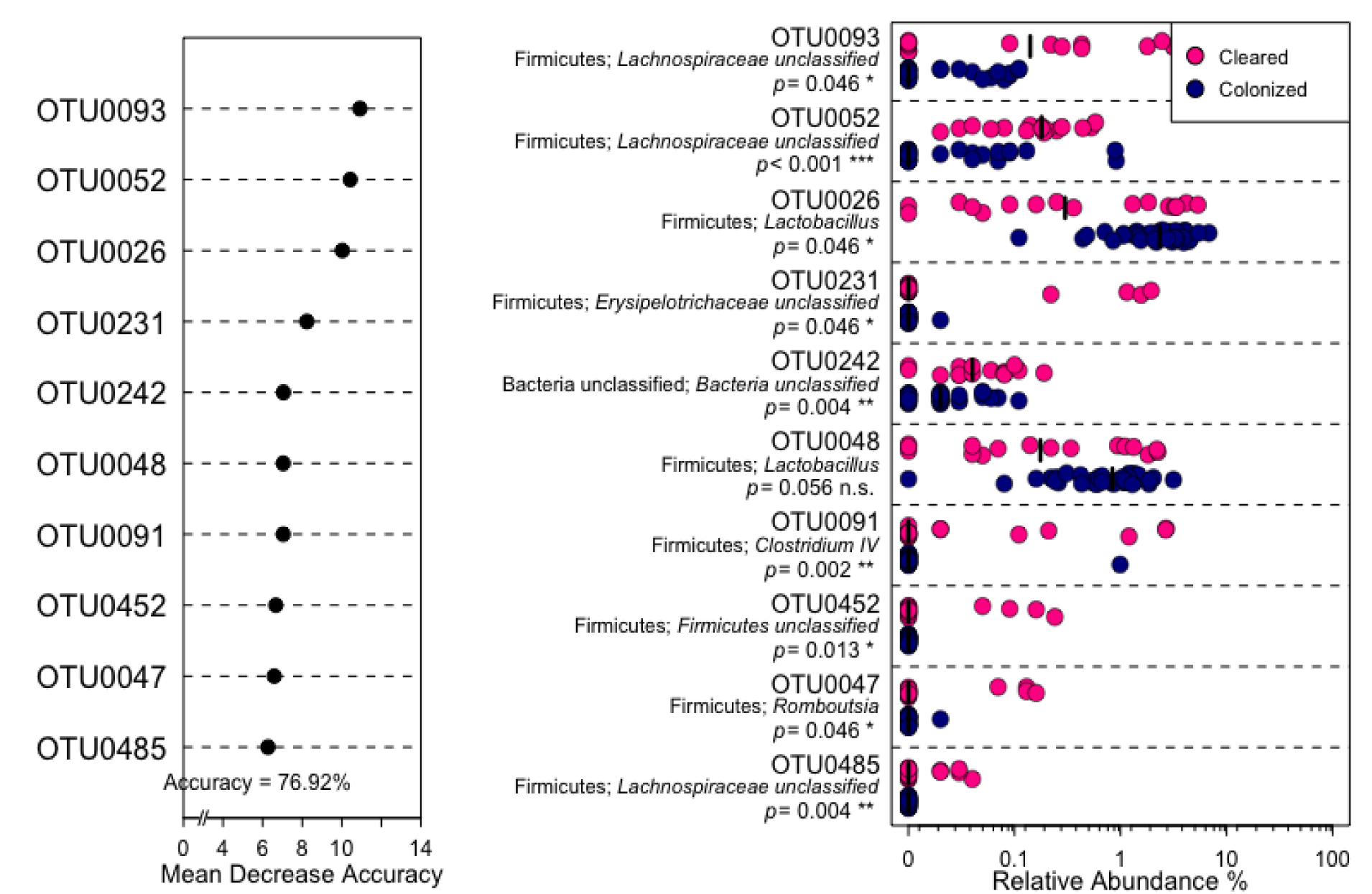

Supplement: FIG S3 [file mSphereDirect.00698-18-sf003.tif]

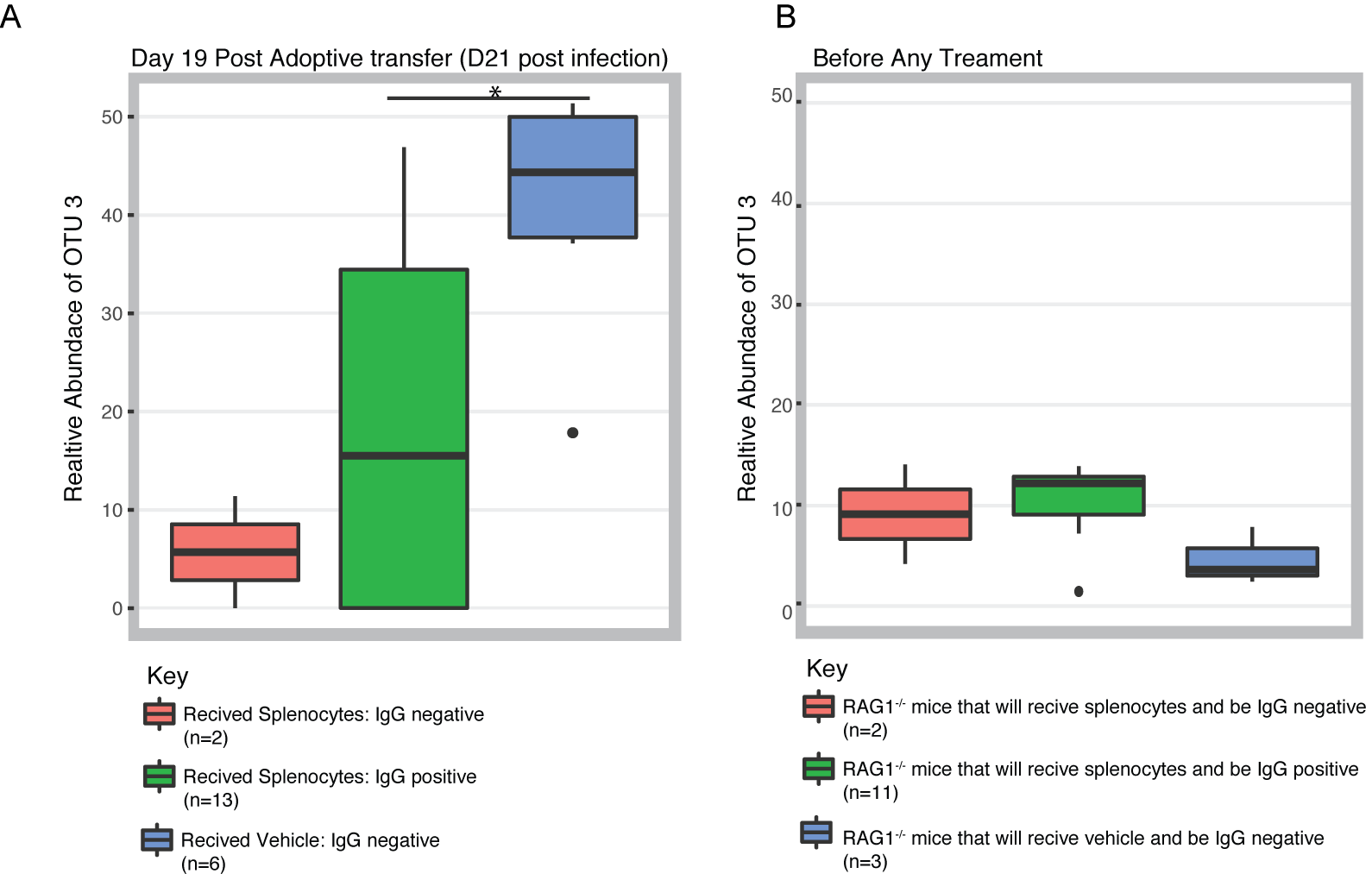

Supplement: FIG S4 [file mSphereDirect.00698-18-sf004.tif]
